# Improvement of *Bacillus subtilis* PI agarase production, hydrolysate scavenging capability assessment, and saccharification of algal biomass for green ethanol generation

**DOI:** 10.1038/s41598-024-65736-4

**Published:** 2024-07-16

**Authors:** Doaa A. Goda, Nagham H. Shalaby, Nadia A. Soliman

**Affiliations:** 1 Bioprocess Development Department, Genetic Engineering and Biotechnology Research Institute (GEBRI), City of Scientific Research and Technological Applications (SRTA-City), New Burg El-Arab City, Universities and Research Institutes Zone, Alexandria, 21934 Egypt; 2 Genetics Department, Faculty of Agriculture (El-Shatby), Alexandria, Egypt

**Keywords:** Agarase, Oligosaccharides, Experimental-design, Bioethanol, Antioxidant, Environmental biotechnology, Microbiology

## Abstract

The goal of the current work was to optimize the growth parameters needed to manufacture agarase enzyme from a non-marine PI strain of *Bacillus subtilis* on an agar-based medium. Using Plackett–Burman design (PBD), nine process parameters were evaluated, and agar, peptone, and yeast-extract were identified as the most significant independent factors influencing agarase production with confidence levels more than 90%. To evaluate the optimal concentrations of the indicated process parameters on agarase production, the Box–Behnken design (BBD) was applied. After optimization*, B. subtilis* strain PI produced 119.8 U/ml of agarase, representing a 1.36-fold increase. In addition the agar hydrolysate fermented products contain the liberated oligosaccharide acts as strong antioxidant which has 62.4% scavenging activity. Also, the agarase yields increased (1141.12, 1350.253, 1684.854 and 1921.863 U/ml) after substitution the agar with algal biomass of *Carolina officinalis* at different concentrations (2, 5, 10 and 15%), respectively. After completing the saccharification process, the resulted hydrolysate was used to produce ethanol through fermentation with *Pichia pastoris* yeast strain as an economical method giving yields (6.68317, 7.09748, 7.75648 and 8.22332 mg/ml), that are higher than using yeast extract peptone dextrose (YPD) medium (4.461 mg/ml).

## Introduction

Agar is the main polysaccharide of the cell walls of red algae (Rhodophacea), and microbial agarases are essential for recycling of red algal biomass^1^. Discovering agar hydrolyzing enzymes helped in studying the structure of agar^2^. Agar is highly heterogeneous in nature with varying amounts of agarose and agaropectin the two main components of agar^3^. A definition for agar should include the name of the algae from which it was prepared; it is species-dependent^4^. Most commercial agar is a blended mixture of agars extracted from different species of red seaweeds^2^.

Agarases could effectively degrade agarose into two categories of oligosaccharides: Agarooligosaccharides and neoagarooligosaccharides by α- and β- agarases, respectively^5^. Oligosaccharides derived from agar hydyrolysis have a wide range of applications; in the health-food, pharmaceutical, and cosmetic industries^6^. Agaro-oligosaccharides can be prepared by acid treatment or by using α-agarases. However, neoagaro-oligosaccharides can be obtained only by β-agarase enzymes^7^. To completely hydrolyze agar into monomers, an agarolytic bacterium capable of assimilating agar as a carbon source often creates a mixture of agarases^8^. Agarase enzymes play important role in various preparative process; in the extraction of labile substances with biological activities such as vitamins, unsaturated fatty acids, and carotenoids^9^. In the preparation of protoplasts^10^ which are useful experimental materials for physiological and cytological studies. Agarase enzymes have been used to recover DNA bands or other biological molecules embedded in agarose gels^11^. They could also be used to prevent biofouling and for the treatment of biofouled surfaces^3^. Furthermore, it is widely used as a food ingredient and is generally recognized as safe (GRAS) food additive by the FDA^12^. It is also used as solidifying agent in microbiological studies^13^. Additionally, agaro-oligosaccharides demonstrate antioxidative properties by scavenging hydroxyl free radicals, superoxide anion radicals, and preventing lipid peroxidation. The oligosaccharides with the sulphate group or with larger molecular masses have better antioxidative effects than those without the sulphate group or with smaller molecular weights^14^.

A small number of agarases are very productive and have superior qualities like high specific activity, good temperature stability, and pH stability. So, efforts are undertaken towards finding more agarases with high activity, various optimum activity conditions and promising stability to shed light on their broadly potential uses. In addition more attention directed to optimize the enzyme production by *Microbacterium* spp., *Bacillus subtilis*, *Dendryphiella arenaria & Agarivorans albus* YKW-34 and then finding the finest condition to maximize the yields through OVAT or experimental design approaches as described by many researchers^15–18^, respectively. Microorganisms that hydrolyze agar can be employed as biological tackles for bioremediation and ecosystem self-cleaning^19^.

Red algae can be utilized as sustainable and renewable biomass for the production of biofuels such as ethanol and butanol, through complete hydrolysis of agar into monomeric sugars and subsequent utilization of glucose and lactic acid by fermenting bacteria and yeasts^6^. Algae saccharification was applied by using agarases from a bacterial isolate *Priestia megaterium* AT7^20^ and marine fungal strain *Dendryphiella arenaria*^16^.

Agarase producing microorganisms include bacteria, actinomyces and marine mollusks. Most agar degrading bacteria have been isolated from marine habitats including seawater, marine sediment, sea sand, red algae, and abalone gut^9^. There are reports that agarases have been isolated from soil, freshwater, and plant rhizospheres, e.g. *Bacillus agar-exedens* from soil^21^, *Cytophaga flevensis* from water^22^, and *Paenibacillus* sp. from plant rhizopheres^23^. A psychrophilic agarase producing strain *Pseudoalteromonas* sp. NJ21 was isolated from antarctic marine environment^24^. Agarase producing bacteria were also isolated from laboratory wastes^18^, some factories^25^, a sewage treatment plant^26^ and drainage wastewater^19^. Research on these non-marine, agar-degrading isolates may provide vision into the ecology of these kinds of microbes^23^.

**Figure 1:**
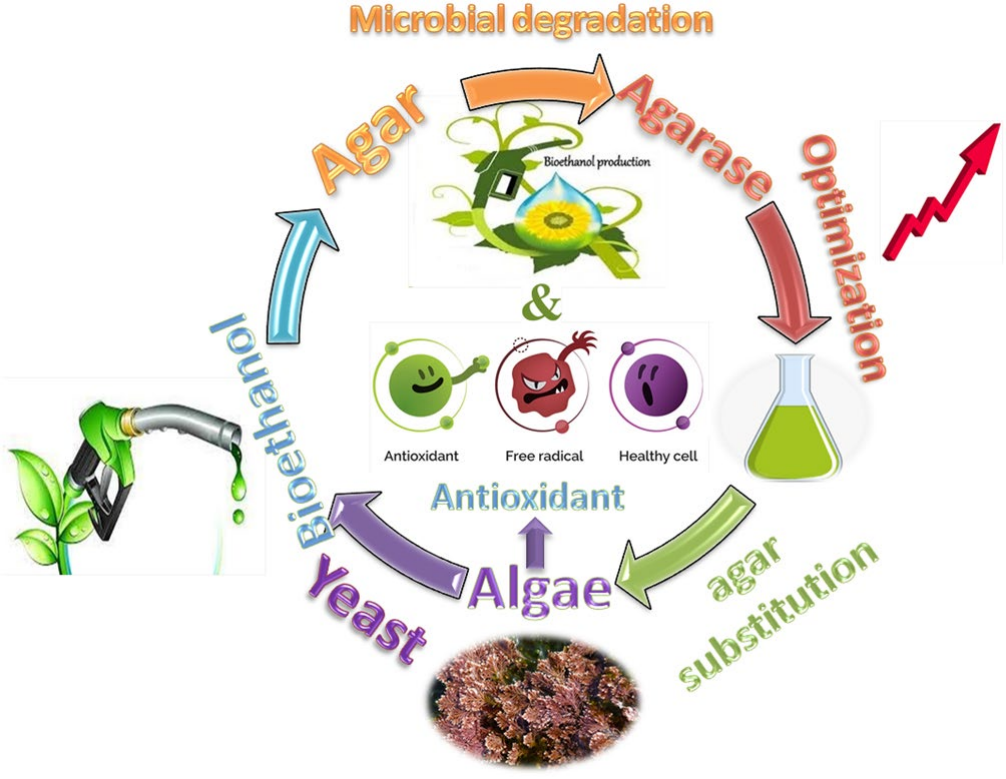
Schematic diagram illustrating agarase optmization from non-marine isolate the antioxidant activity of agar oligosaccharides evaluation, and the bioethanol generation.

Thus, the focus of our work was on the optimization and synthesis of agarase from non-marine isolate. Subsequently, focus on evaluating the antioxidant activity of agar oligosaccharides and the techno-economical synthesis of bioethanol exploiting saccharified algal biomass. Figure 1 provided a summary and full illustration of these steps.

## Results

### Statistical optimization for agarase production from of *Bacillus* subtilis PI

#### Plackett–Burman screening design

The PB experimental design is a potent tool for analyzing and examining the crucial factors affecting the response. The factors applied in PB design were selected based on many preliminary experiments (data not shown). The results of PB presented in Table 1 show that the agarase activity varied greatly among the 14 trials from 11.43 to 87.70 U/ml, indicating that medium adjustment is crucial to achieving a high agarase output. The investigation of multiple regression revealed an association between the generation of agarase and the nine independent factors. The regression analysis presented in Table 1 indicates that the factors (agar, peptone, yeast-extract, NaNO_3_, and K_2_HPO_4_) had a favorable impact on agarase activity, while it was noticed that MgSO_4_, FeSO_4_, pH, and temperature all had a deleterious impact.

**Table 1:** Randomized PB matrix designed for evaluating factors influencing agarase production by *Bacillus subtilis* PI and Statistical analysis of PBD.

Trials	Variables									Agarase (U/ml)		
	A	B	C	D	E	F	G	H	I	Experimental	Predicted	Residual
1	1	− 1	− 1	1	− 1	1	1	1	− 1	32.44	30.92	1.52
2	− 1	1	− 1	− 1	1	− 1	1	1	1	25.37	23.86	1.52
3	1	1	− 1	− 1	− 1	1	− 1	− 1	1	49.73	48.38	1.35
4	1	− 1	1	1	1	− 1	− 1	− 1	1	77.03	75.51	1.52
5	− 1	1	1	1	− 1	− 1	− 1	1	− 1	61.44	60.09	1.35
6	− 1	− 1	1	− 1	− 1	1	− 1	1	1	36.90	36.34	0.5588
7	1	− 1	− 1	− 1	1	− 1	− 1	1	− 1	35.84	35.45	0.3882
8	− 1	− 1	− 1	1	− 1	− 1	1	− 1	1	11.43	11.04	0.3882
9	− 1	1	− 1	1	1	1	− 1	− 1	− 1	28.72	28.16	0.5588
10	1	1	1	1	1	1	1	1	1	84.18	83.79	0.3882
11	− 1	− 1	1	− 1	1	1	1	− 1	− 1	44.52	43.17	1.35
12	1	1	1	− 1	− 1	− 1	1	− 1	− 1	87.70	87.14	0.5588
13	0	0	0	0	0	0	0	0	0	46.38	46.99	− 0.6075
14	0	0	0	0	0	0	0	0	0	36.16	46.99	− 10.83

Variables (% w/v)	Code	Coded and actual levels (%) actual levels		Statistical analysis of PBD showing *coefficient* values, main effect, *t* and *p* values, and *confidence level* % for each variable affecting agarase production								
		− 1	1	*Coefficients*	*Main effect*	*Standard Error*	*Confidence level (%)*					
model				46.99		1.53	99.56					
Agar	A	0.1	0.5	13.21	26.42	1.65	99.87					
Peptone	B	0.2	1	8.25	16.5	1.65	99.24					
Yeast extract	C	0.2	2	17.35	34.7	1.65	99.95					
NaNO_3_	D	0.01	0.1	1.26	2.52	1.65	51.28					
K_2_HPO_4_	E	0.01	0.1	1.34	2.68	1.65	53.51					
MgSO_4_	F	0.01	0.1	− 1.86	− 3.72	1.65	67.61					
FeSO_4_	G	1 mg	5 mg	− 0.3335	− 0.667	1.65	14.99					
Temp	H	30	37	− 1.91	− 3.82	1.65	68.8					
pH	I	6.0	8.0	− 0.5010	− 1.002	1.65	22.29					

To examine the correlation among the parameters at a 90% or greater confidence level, the *p* value via the analysis of variance (ANOVA) for every response was identified. The results indicate a statistically significant relationship between the parameters at a 95.56% confidence level, according to the ANOVA test, which yields *p* = 0.0044. The *R*^2^_Predicted_ of 0.9163 is in reasonable agreement with the *R*^2^_Adjusted_ of 0.9370; i.e. the difference is less than 0.2. The signal-to-noise ratio is measured by the appropriate (adequate) precision value. A number greater than 4 is preferred, as it signifies the model's good fit. The current model has a sufficient precision value of 15.706, which means that it can be utilized to explore the design space. The results of Table 2 indicate that the model has standard deviation, mean, PRESS, and coefficient of variation (C.V. %) values of 5.73, 46.99, 567.79, and 12.20, respectively.

**Table 2:** Fit Statistics and ANOVA for selected factorial model.

Fit statistics						
Std. Dev	5.73	R^2^	0.9806			
Mean	46.99	Adjusted R^2^	0.9370			
C.V. %	12.20	Predicted R^2^	0.9163			
PRESS	567.79	Adeq Precision	15.7065			

ANOVA for selected factorial model						
Source	*Sum of Squares*	*df*	*Mean Square*	*F-value*	*p value*	
Model	6654.21	9	739.36	22.50	0.0044	significant
Agar	2093.99	1	2093.99	63.72	0.0013	
Peptone	816.44	1	816.44	24.85	0.0076	
Yeast extract	3613.39	1	3613.39	109.96	0.0005	
NaNO3	19.20	1	19.20	0.5843	0.4872	
K_2_HPO_4_	21.40	1	21.40	0.6513	0.4649	
MgSO_4_	41.51	1	41.51	1.26	0.3239	
FeSO_4_	1.33	1	1.33	0.0406	0.8501	
Temp	43.93	1	43.93	1.34	0.3120	
pH	3.01	1	3.01	0.0917	0.7771	
Residual	131.44	4	32.86			
Cor Total	6785.65	13				

Equation (1) illustrates the linear equation that was generated to reflect the generation of agarase in terms of the independent factors under investigation. This was found to be an excellent fit based on the resultant *R*^2^ = 98%, since only about 2.0% of the total variances caused by the factors did not suit the expected agarase activity (Table 2).

1 $$\begin{aligned} {\text{Y}}_{{{\text{agarase}}}} & = {46}.{98689} + {13}.{2}0{\text{981A}} + {8}.{\text{24842B}} + {17}.{\text{35269C}} \\ & \quad + {1}.{2649}0{\text{D}} + {1}.{\text{33551E}} - {1}.{\text{85993F}} \\ & \quad - 0.{\text{333469G}} - {1}.{\text{91325 H}} - 0.{5}0{1}0{\text{45I}} \\ \end{aligned}$$

**Figure 2:**
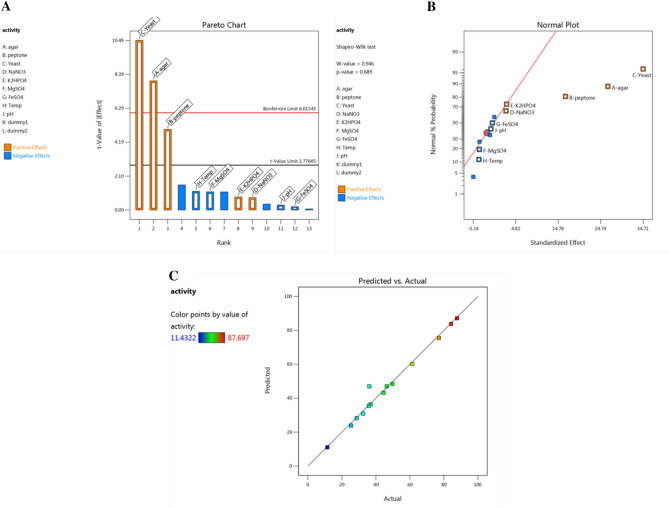
Plackett–Burman design (PBD): (**A**) Pareto chart illustrating the order and signifcance of the factors affecting agarase production by *Bacillus subtilis* PI strain, (**B**) Normal probability plot (NPP) of the residuals to assess whether the model is adequate, and (C) Plot of predicted versus actual results of agarase production.

The Pareto chart (Fig. 2A) displays the relative importance of the numerous factors influencing the synthesis of agarase. It showss that the three most important factors influencing agarase production using *Bacillus subtilis* PI are yeast-extract, agar, and peptone. This result has been confirmed by normal probability plot of the standardized effect where it determines the direction, magnitude, and significance of each factor. At the 0.05 level, the major impacts of components yeast-extract, agar, and peptone on this plot are statistically significant. Furthermore, the effect's direction is indicated by the plot. These three factors have a standardized favorable effect. Agarase activity rises when the factor's low level is changed to a high level during the process (Fig. 2B). The end products of the agarase generating experiment indicate a significant correlation among the real and forecast values, as indicated by the *determination coefficient R*^2^ of 0.98 (Fig. 2C).

Three verification experiments were used to streamline the medium and focus mostly on agar as the media backbone. In the first, the usual medium and conditions suggested by the PB equation model were employed; in the second, NaNO_3_, K_2_HPO_4_ and MgSO_4_ were excluded; and in the third none of the following were present, NaNO_3_, K_2_HPO_4_, MgSO_4_ and FeSO_4_. According to the results, the agarase activity yields from the first and second experiments were about equal, whereas the third experiment produced a reduced activity. Consequently, in the next experiment, NaNO_3_, K_2_HPO_4_, and MgSO_4_ were expunged from the medium.

### Response surface methodology (Box–Behnken design)

The optimal response zone for agarase synthesis in terms of activity (U/ml) was discovered using Box–Behnken design (BBD). Finding significant factors, assessing their correlations, and forecasting the outcome are made easier with the use of the Response Surface Methodology (RSM) regression analysis. BBD ascertained the ideal concentrations of three factors: agar (A), peptone (B), and yeast-extract (C) along with the interactions between them for agarase synthesis.

Factors that have a beneficial impact on agarase production have been employed at high level, whereas the factors that negatively affecting agarase production are kept at low level for additional improvement by BBD. Using a multiple regression analysis approach, the three tested factors with fourteen trails were examined, and the appropriate % confidence rates were ascertained (Table 3). In the BB experiments, variance analysis was carried out using ANOVA testing, and the findings are reported in Table 4. The recorded response (agarase activity), and the investigated factors have a statistically significant association, as indicated by the derived result of *p* = 0.0351. Based on statistical evaluation, the model is extremely significant (low *P*-value, 0.0351 < 0.05) for agarase synthesis. The significance values show that the quadratic effects and linear coefficients of agar (A), peptone (B), and yeast-extract (C) are significant (Table 4).

**Table 3:** BB designed matrix for the selected 3-variables influencing agarase production by *Bacillus subtilis* PI.

Trial	Variables			Agarase (U/ml)(		
	A	B	C	Experimental	Predicted	Residual
1	− 1	− 1	0	77.79	75.06	2.74
2	1	− 1	0	80.03	85.23	− 5.20
3	− 1	1	0	103.82	98.62	5.20
4	1	1	0	100.47	103.21	− 2.74
5	− 1	0	− 1	68.69	72.47	− 3.78
6	1	0	− 1	85.42	81.26	4.16
7	− 1	0	1	94.52	98.68	− 4.16
8	1	0	1	108.44	104.66	3.78
9	0	− 1	− 1	64.22	63.18	1.04
10	0	1	− 1	92.29	93.71	− 1.42
11	0	− 1	1	99.17	97.75	1.42
12	0	1	1	107.72	108.77	− 1.04
13	0	0	0	95.27	94.06	1.21
14	0	0	0	92.85	94.06	− 1.21

Variables (% w/v)	Code	Coded level and actual level				
		− 1	0	1		
Agar	A	0.3	0.6	0.9		
Peptone	B	0.7	1.2	1.7		
Yeast extract	C	1.5	2	2.5

**Table 4:** Statistical analysis of BB design showing *coefficients, t –*and* p-values* for significant variables affecting on agarase production by *Bacillus subtilis* PI .

Fit statistics									
Std. Dev	5.95	R^2^	0.943						
Mean	90.77	Adjusted R^2^	0.8147						
C.V. %	6.55	Adeq Precision	9.0701						

ANOVA for quadratic model						Coefficients in terms of coded factors			
Source	Sum of Squares	*df*	Mean Square	F- value	*p* value	Coefficient estimate	Standard Error	95% CI high	
Model	259.9	9	2339	7.350	0.0352	94.06	4.20	105.7	significant
A-Agar	109.0	1	109.0	3.080	0.1540	3.690	2.10	9.53	
B-Peptone	863.0	1	863.0	24.41	0.0078	10.39	2.10	16.2	
C-Yeast extract	1231	1	1231	34.82	0.0041	12.40	2.10	18.2	
AB	7.770	1	7.770	0.2199	0.6635	− 1.390	2.97	6.86	
AC	1.980	1	1.980	0.0560	0.8246	− 0.7034	2.97	7.55	
BC	95.24	1	95.24	2.690	0.1761	− 4.880	2.97	3.38	
A^2^	20.96	1	20.96	0.5927	0.4843	− 2.560	3.32	6.67	
B^2^	3.030	1	3.030	0.0856	0.7844	− 0.9727	3.32	8.26	
C^2^	15.97	1	15.97	0.4516	0.5384	− 2.230	3.32	7.00	
Residual	141.4	4	35.36						
Lack of Fit	138.5	3	46.17	0.1823	15.81				
Pure Error	2.920	1	2.920						
Cor Total	2480.5	13							

The second-order polynomial model equation (Eq. 2) has been proposed to examine the relationship between various factors and find the greatest amount of agarase production that corresponds to the ideal concentration of agar, peptone, and yeast-extract. The ideal concentrations of the independent factors (X_agar_, X_peptone_, and X_yeast-extract_) determine the maximal agarase production with predictability:

2 $$\begin{aligned} {\text{Y}} & = {94}.0{5999} + {3}.{\text{69137A}} + {1}0.{\text{38657B}} + {12}.{4}0{\text{493C}} \\ & \quad - {1}.{\text{39417 A}}*{\text{ B}} - 0.{7}0{\text{3438A}}*{\text{C}} - {4}.{\text{87959XB}}*{\text{C}} \\ & \quad - {2}.{\text{55915XA}}^{{2}} - 0.{\text{972742B}}^{{2}} - {2}.{\text{23385C}}^{{2}} \\ \end{aligned}$$

### Verifying the accuracy of the model

**Figure 3:**
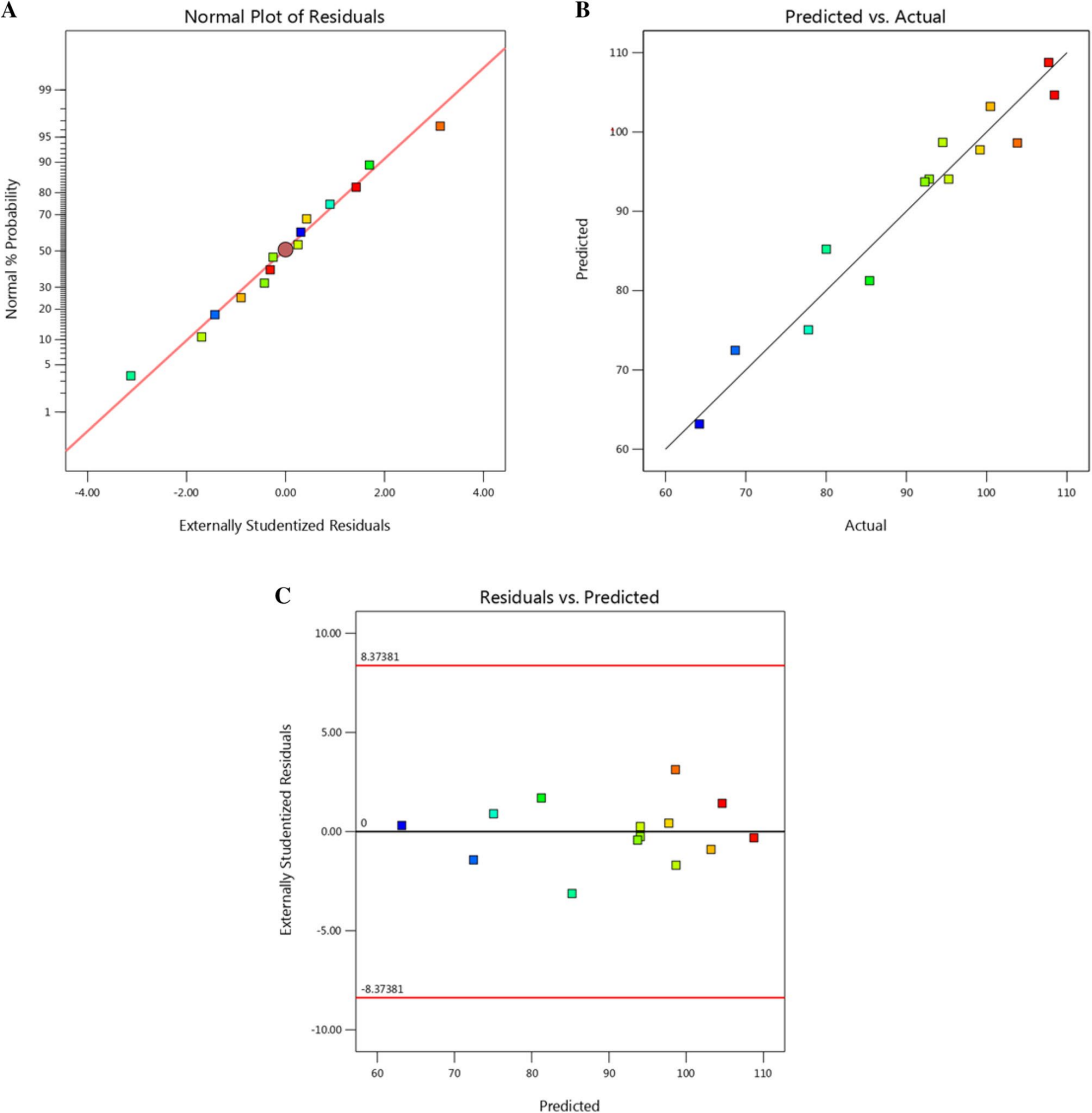
Box-Behnken design: (**A**) Normal probability plot (NPP) of the residuals to assess whether the model is adequate, (**B**) plot of predicted versus actual results of agarase production, and (**C**) Internally studentized residuals versus predicted agarase production.

In order to confirm the accuracy of the methodology, certain statistical analyses were performed. One essential graphical method for observing the residuals' distribution and assessing the suitability of the model is the normal probability plot (NPP) of the residuals. The variance between the practical findings and the expected response values of the theoretical model is known as the residual. A small residual value indicates that the model matches the experimental data well and that the model prediction is quite accurate. The NPP of the studentized residuals is plotted against the response data estimated by the model in Fig. 3A. The study's normally distributed residual points are situated adjacent to the diagonal straight line, demonstrating the model's compatibility with the experimental findings of the agarase production. The residuals are not regularly distributed if they deviate from this straight line.

The model's accuracy is confirmed by the significant correlation found between the anticipated outcomes and the experimental findings of agarase synthesis, as shown in Fig. 3B, which shows a plot of predicted against real agarase production with points close to the fitted line. Figure 3C displays a plot of the estimated agarase output against the studentized residuals. The residuals' uniform and random distribution above and below the zero line, devoid of any discernible pattern, shows that the residuals' variance is constant and validates the accuracy of the model.

**Figure 4:**
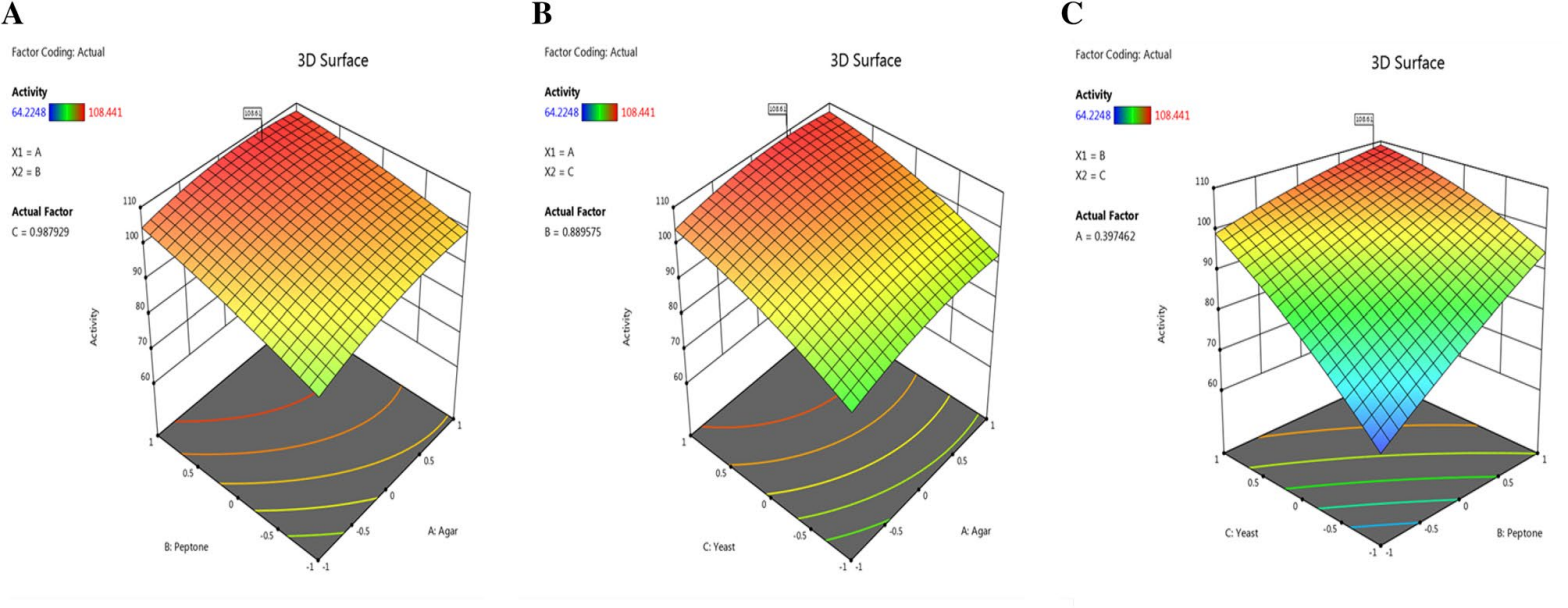
A 3D response surface and contour graphs illustrate the effects of agar (**A**), peptone (**B**), and yeast extract (**C**) on agarase production.

Furthermore, Fig. 4 displays, the three-dimensional graphs produced by the design expert program, the simultaneous impacts of the three investigated independent factors on the agarase activity. The maximum polynomial model point yielded the ideal values for the three factors under study, agar (0.411), peptone (0.903), and yeast-extract (0.959). The anticipated estimated agarase activity for these factors was determined to be 108.6 U/ml. A Y value of 119.8 U/ml was achieved by the bench scale confirmatory test, meaning that the estimated accuracy of the model was 110.3%. According to the BB experiment, the following circumstances resulted in the greatest amount of agarase activity (g%): 0.791; agar, 1.645; peptone, 24.494; yeast-extract, Fe_2_SO_4_; 5 mg%, pH; 6, and a cultivation temperature of 30 °C, with a measured agarase activity equal to119.8 U/ml.

**Figure 5:**
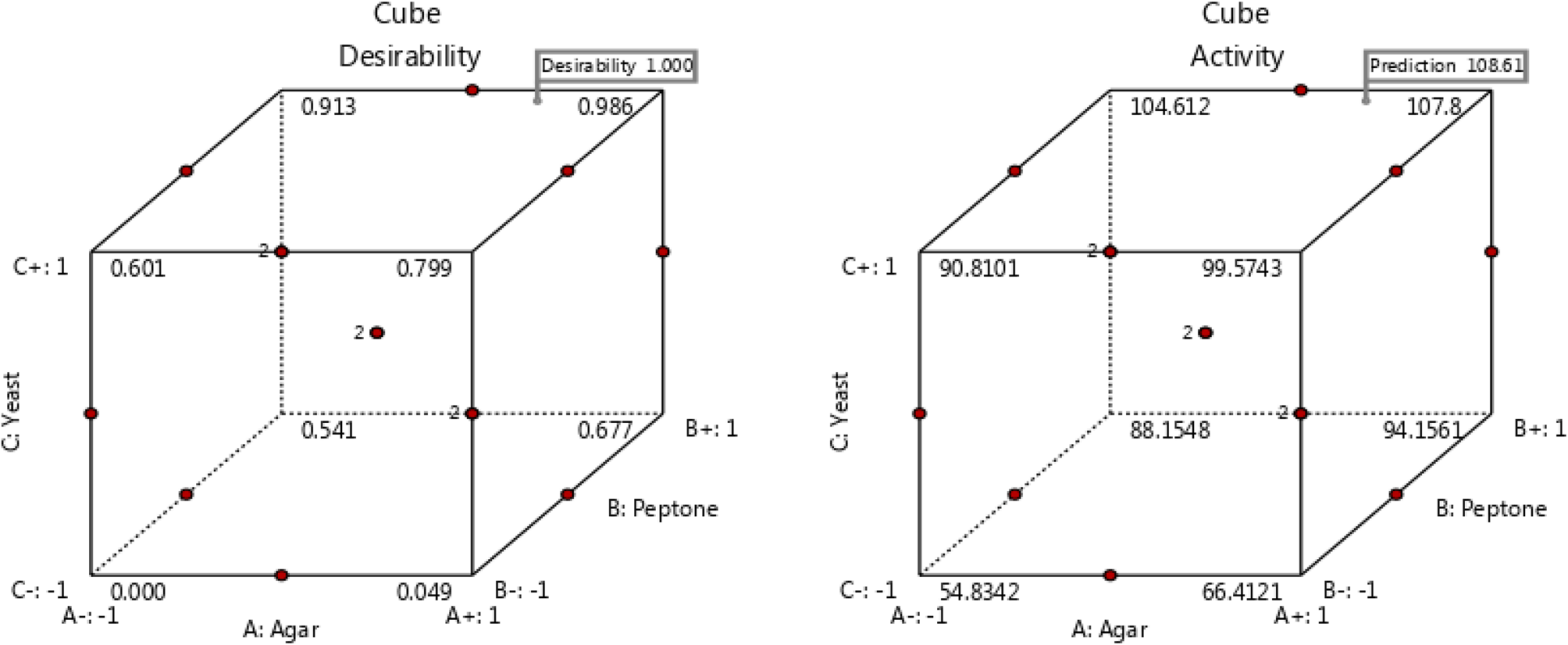
Cube plot of desirability (left), and activity (right) illustrates how three interconnected factors (**A**: agar, **B**: peptone, and **C**: yeast extract) influence the agarase activity (**C**).

Cube Plot for agarase activity and its desirability (Fig. 5), this plot illustrates the expected response (agarase activity) as a function of the three significant factors: agar (A+), peptone (B+), and yeast-extract (C+). It also demonstrates how these three factors interact to affect the response. Because all of the values displayed are predictions, charts can be created even in cases where actual data is unavailable. The filtration rate is maximal at settings agar (A+), peptone (B+), and yeast-extract (C+), with a predicted response of 108.4, which also corresponds to the highest activity.

### FTIR investigation of the chemical profile of agar prior to and following biological treatment

**Figure 6:**
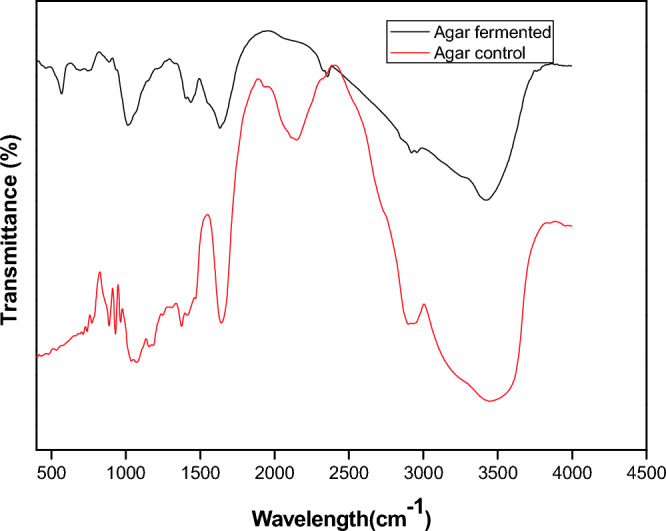
FT-IR spectra of agar control (red) and agar fermented (black).

FTIR was employed for agar and fermented agar (Fig. 6) in which the band at roughly 1640 cm^−1^ suggests the probable existence of a sugar ring. The pronounced peaks prior to and following biological treatment at 3446 cm^−1^ and 3421 cm^−1^, respectively, illustrate the stretching vibration of OH, whereas after pretreatment, the peak shrinks, indicating the hydrolysis of agar. According to the fermented agar's spectrum, the –CH and –CH_2_ groups are responsible for the peak, which is located at 2920 cm^−1^. Additionally, the formation of conjugated peptide bonds is indicated by a spectrum at 1631 cm^−1^, which is ascribed to the N–H and C=O stretching groups, whereas the peak 1013 cm^−1^ is connected to the presence of carbohydrates stretching vibration of C–O. The occurrence of a 3, 6-anhydrous galactose linkage is indicated by its peak at 933 cm^−1^.

### Evaluation of the antioxidant activity of the enzyme hydrolysate

The antioxidant potential of the oligosaccharides in the hydrolyzed agar was investigated, and the agar-hydrolysate's capacity to scavenge free radicals was measured using the 1, 1-diphe-nyl-2-picrylhydrazyl (DPPH). The free radical scavenging activity (RSA) % of the hydrolysate at different time (0, 15, 30 and 45 min) was (22.1 ± 0.65, 53.4 ± 0.83, 60 ± 0.54, and 62.4 ± 0.32%), respectively, whereas its value for ascorbic acid (control) was (54.6 0.62 ± , 93 ± 0.43, 93.8 ± 0.21, and 90.44 ± 0.45%), respectively and RSA % of hydrolysate after algae substitution (15%) was 98 ± 0.28%).

### Green conversion of algal biomass to bioethanol

After the fermentation process by *Bacillus subtilis* PI (optimization of agarase production using agar as a substrate), the agar was substituted with biomass of *Carolina officinalis* with different concentrations (2%, 5%, 10% and 15%) in the same optimized medium but with algal biomass instead of agar, the agarase yields increased (1141.12, 1350.253, 1684.854 and 1921.863 U/ml), respectively compared to its values by using agar as asubstrate (119.8 U/ml).

**Figure 7:**
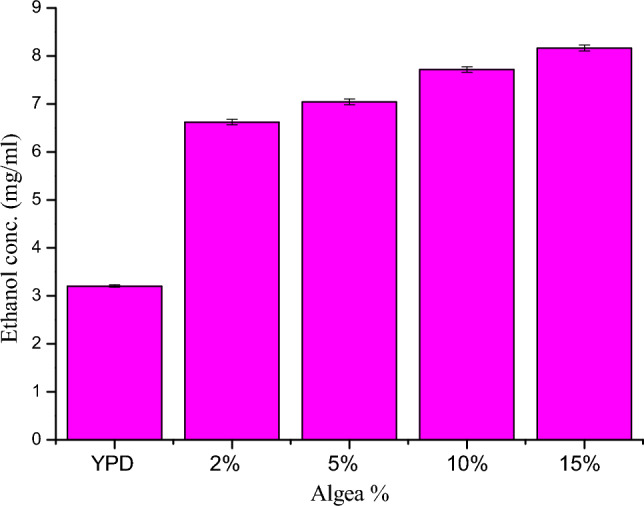
Ethanol production conc. (mg/ml) by using different conc. of algal biomass (2, 5, 10, and 15%) in agarase optimized medium compared with its production in YPD medium without algal biomass (control).

For ethanol production process (sterile fermentation hydrolysate resulted from fermentation of algae inoculated with yeast (*Pichia pastoris*), the result of this experiment indicated that as concentration of algal biomass increase (2%, 5%, 10% and 15%), the bioethanol production increased comparing with ethanol production by yeast in YPD medium, where it was in (mg/ml) 6.68317, 7.097, 7.756, 8.223 and 4.461, respectively (Fig. 7).

## Discussion

The enzymes known as agarases are responsible for catalyzing the decomposition of agar. Agarases are widely used in the cosmetics, food, and medicinal industries because they generate oligosaccharides with intriguing properties^9^.

In this regard, this study has concentrated on the synthesis and development of agarase in addition to the generation of bioethanol from the destruction of algal biomass by the effect of agarase. Two phases (PB and BB) of a sequential optimization technique were used to increase *Bacillus subtilis* PI's agarase output.

In most cases, it is crucial to evaluate as many factors as possible and to understand their significance when determining which ones are influencing the development of particular secondary metabolites^27^. A good, speedy screening procedure and mathematical calculation of the importance of several factors in one experiment are provided by the PB design, which saves time and maintains valid data about each factor. Examining the interactions between thus many factors is not the screening program's top goal, even though interaction is missing in this model. The PB results in this investigation ranged widely, from 11.43 to 87.70 U/ml of agarase. This variant illustrates how crucial medium tuning is to achieving increased output.

The most important factors raising agarase production, as indicated by the PB results, were agar, peptone, and yeast-extract, with p values of 0.0013, 0.0076, and 0.0005, respectively while, NaNO_3_ and K_2_HPO_4_ did not substantially alter agarase productivity, this in agreement with Fu et al.^15^ who reported that the use of yeast extract as nitrogen source produced the highest agarase activity. It was also stated that the use of organic nitrogen sources was preferable to inorganic ones for agarase synthesis. Consequently, it was discovered that nitrogen was a necessary component for the agarolytic enzyme's synthesis. Similar findings on this impact have been documented in studies that used yeast extract as a source of nitrogen^28^.

However, our results are consistent with those of Lakshmikanth et al.^28^, who found that agarase synthesis was best suited with agar as the carbon source.

According to our result temperature and pH have a negative effect on agarase production, the obtained conclusion was contrasted with another *Bacillus* sp. agar-degrading bacterial agarase, which had an ideal pH of 7.6^29^. One benefit of the PBD is that, regardless of the factor's type (nutritional or physical) or the signs' nature (positive or negative); operators can rate the impact of numerous factors on the recorded response^30^.

The second stage of optimizing agarase synthesis involves measuring the constituents using statistical methods like RSM, an effective experimental approach for maximizing agarase production conditions. RSM's primary benefit is that it just requires a few experimental runs to gather sufficient data for conclusions that are statistically significant. It is quicker, requires less time and effort, and avoids misconceptions that can arise from using the traditional way^30^.

The model was extremely significant as shown by the F value of 7.35 and the *p* value of 0.0352 for the *Bacillus subtitles* PI. The response's actual and projected values were similar, as shown by *R*^2^_correlation coefficient_ value of 0.9430, in line with the findings from Maharjan et al.^31^ who said that a higher *R*^2^ values support the model. The model's assurance in ideal conditions is demonstrated by the substantial amount of accuracy, as the expected estimated agarase activity from BBD was found to be 108.6 U/ml and the laboratory scale confirmatory evaluation produced a Y value of 119.8 U/ml.

Furthermore, the BBD agarase activity value was 1.366 times higher than the PBD activity; this demonstrated why the optimization process is important and necessary. Our findings align with^30,32,33^, wherein the RSM is recognized as a contemporary statistical method for optimizing experimental settings and resolving analytical issues. RSM assists in determining the most productive factors to examine interactions with, figuring out the ideal quantity of each factor, and guaranteeing maximum output in a predetermined number of trials^30,33^.

In order to ascertain whether chemical structural alterations were present in the agar and the byproducts produced by the fermentation of agar, Fourier transform infrared spectroscopy (FTIR) was employed.

In agreement to El-Hefan et al.^34^ the peak, which is located at 2920 cm^−1^ for fermented agar indicated the –CH and –CH_2_ groups and the formation of conjugated peptide bonds is indicated by a spectrum at 1631 cm^−1^, which is ascribed to the N–H and C=O stretching groups, whereas the peak 1013 cm^−1^ is connected to the presence of C–O group.

Our study concurring with some researchers^35,36^ reported that the peak at 930 cm^−1^ indicated the occurrence of a 3, 6-anhydrous galactose linkage, which confirms the chemical nature of agar.

However, our results are consistent with this of Lyons et al.^37^ who found that, the pronounced peaks prior to and following biological treatment at 3446 cm^−1^ and 3421 cm^−1^, respectively, illustrate the stretching vibration of OH and suggest the occurrence of the hydroxyl group in agar; in contrast, the peak diminishes via biological treatment, signifying the agar's decomposition.

The results of this investigation indicated the significant antioxidant capacity of the polysaccharides generated by hydrolysis of agar using agarase developed from *Bacillus subtilis*. It has been revealed that agar derived oligosaccharides have great commercial value owing to their biological and physiological characteristics. In alignment with these results, Zhang et al.^38^ revealed that oligosaccharides produced from the hydrolysed agar by agarase of *Vibrio natriegens* had remarkable scavenging action. Similar to this, oligosaccharides made by enzymatically treated agar using the agarase originated from *Stenotrophomonas* sp. NTA, shown strong antioxidative effectiveness by inhibiting the ABTS (2,2′-azino-bis-(3-ethylbenzothiazoline-6-sulfonic) acid) radicals, hydroxyl and DPPH^39^.

After the optimization process of agarase production, agar in the final optimized medium was substituted with dried *Carolina officinalis* biomass at different concentration (w/v %) 2, 5. 10 & 15 for the agarase production using algae instead of agar as a substrate, where the fermentation optimization medium composed of: 1.645; peptone (g%), 24.494; yeast-extract (g%), Fe_2_SO_4_; 5 mg%, pH; 6, and a cultivation temperature of 30 °C to induce agarase production using *Bacillus subtilis* strain.

The global populace is still growing, which raises energy consumption and results in a large and swift reliance on fossil fuels and the rising cost of petroleum-based products combined with worries about national security and the environment has sparked a fervent interest towards researchers in creating commercially feasible methods for producing renewable energy sources for transportation.

Sugar crops and terrestrial starch like sugar cane and corn are the most common feed stocks for the bioethanol generation; nevertheless, the utilization of them for the manufacture of biofuel is limited by the existence of lignin, which is hard to break down^40,41^. In light of this, using alternative non-terrestrial raw materials of which marine macroalgae can be a crucial source appears to be a sustainable and ecological way to meet the global energy need for biofuels^40,42^. The primary factors responsible for increasing the interest in using macroalgae to produce bioethanol are their high carbohydrate content (about 60% w/w)^40,43^, lack of lignin, ability to ferment, ability to grow quickly and easily in a variety of environments, and rising hydrolysis efficiency^40,41^.

In order to address our ever-growing energy needs while safeguarding the environment, we are exploring sustainable, eco-friendly, economical, and high-performing biofuels that emit fewer greenhouse gases^44^. About seventy-five percent of global biofuel use is made right now up of bioethanol^45^. Its feasibility as a sustainable fuel option is confirmed by the billions of liters generated annually at a rapid rate of development of 7%. Consequently, the creation of bioethanol from *Carolina officinalis* biomass was the second goal of our study. As our study indicated that the bioethanol production through fermentation process of 15% red algae yielded 8.22332 mg/ml which is equal to 27.4 mg_bioethanol/_g_biomass algae_ this is in contrast to Pardilhó et al.^46^ who reported that the bioethanol production using marine macoroalgae waste was 21 mg_bioethanol /_g_biomass algae._

## Materials and methods

### Microorganism

The present investigation employed *Bacillus subtilis* PI, a local strain from Egypt that has been recently identified as a producer of agarase and submitted in GenBank under the accession number Ac: MW582095.1.

### Inoculum preparation

Cells of *Bacillus subtilis* PI from a freshly produced plate were allowed to grow in a 30 ml aliquot of YPD broth medium which composed of (g%): yeast extract 1; peptone 2; and dextrose 2, that was dispensed in a 250 ml Erlenmeyer flask as the inoculum. The incubation process was conducted for roughly 24 h at 30 °C and 200 rpm.

### Qualitative and quantitative determination of agarase

Both qualitative as well as quantitative analyses were run to find out whether the agarase enzyme was present in the *Bacillus subtilis* PI culture. The Lugol's iodine solution (1 g Iodine, 2 g KI, and 100 ml distilled water) was used to cover the plate cultures (g%): Yeast extract 1, peptone 2, and 1.5 agar, for the qualitative test. This solution stains the agar into a dark brown color, but it's unable to stain the oligosaccharide of agar that has been broken down^47^. For the quantitative assay, by monitoring the rise in the concentration of reducing sugar, the activity of agarase was ascertained. After adding the 250 µl enzyme to 250 µl of substrate (0.5% agarose in phosphate buffered saline solution (PBS), pH 7.0, the mixture was incubated for 30 min at 60 °C. The enzyme reaction was then stopped by placing the reaction eppendorf in a boiling water bath for 10 min, followed by cooling at ambient temperature. Using a modified method of Gomaa et al.^16^ technique, the reaction mixture was tested for converting sugar as galactose, and then reading the absorbance at 540 nm. Blanks were run concurrently with enzyme and substrate solutions, and the calibration curve was created using galactose solutions at a given concentration. The quantity of enzyme required to create one μmol of galactose in the assay conditions was designated as one unit of agarase activity.

### Production of agarase

The physical and chemical factors for *Bacillus subtilis* PI's agarase production, which uses agar as the base component, have been improved in two stages. The first involved screening physicochemical characteristics using Plackett–Burman design (PBD). The second was to optimize the key factors influencing agarase generation using Box–Behnken design (BBD).

### Plackett–Burman design (PBD)

The design was used to identify the crucial components that had a major impact on the agarase production. After many preliminary experiments the relative significance of nine parameters (agar, peptone, yeast extract, NaNO_3_, K_2_HPO_4_, MgSO_4_, FeSO_4_, temperature, and pH) that affected agarase production was ascertained using a PB experimental design consisting of 14 trials (Two central-point batches and twelve primary batches).

The two widely separated levels of the independent factors a high (+ 1) and low (− 1) were utilized to define the maximum and minimum limits of the ranges that each factor spanned, respectively. Table 1 presents matrix design in addition to the list of the factors that were examined along with their related coded and the real values.

Based on the first-order model Y = β0 + ∑ βi xi, PBD uses Y to denote the response, β0 to describe the equation's intercept, βi to reflect the parameter estimate, and xi to indicate the parameter. The weight of each factor under study was ascertained by computing the *p*-value through the utilization of standard regression analysis. Regression analysis was done using ANOVA, and R and *R*^2^ values were computed to assess the effectiveness and efficiency of the regression model. The factors that substantially affected the agarase production and had confidence levels over 95% and *p* values lower 0.05 (*p* < 0.05) were chosen for additional optimization using BBD^31^.

### Response surface methodology

Following the PBD, the three parameters (agar, peptone, and yeast) with the greatest confidence levels were allowed to be enhanced even more. BBD was used to investigate the response surface's characteristics in the experimental area, assess the optimal values of key factors, and examine the interactions between the factors that were chosen because they had a substantial impact on agarase production. The design matrix with 12 trials along with two core trials to identify human error, the response that was observed (agarase production), and three levels for the chosen parameters, denoted as high (+ 1), medium (0), and low (− 1) all are displayed in Table 3. The model was implemented using the coefficient outcomes of each factor^48,49^.

The agarase production (Y) for the three selected factors was predicted using the following second-order polynomial structured model, which was dependent on the cultivation conditions (A-I):

$$\begin{aligned} {\text{Y}} & = \beta 0 + \beta_{{\text{A}}} {\text{A}} + \beta_{{\text{B}}} {\text{B}} + \beta_{{\text{C}}} {\text{C}} + \beta_{{{\text{AB}}}} {\text{AB}} + \beta_{{{\text{AC}}}} {\text{AC}} \\ & \quad + \beta_{{{\text{BC}}}} {\text{BC}} + \beta_{{{\text{AA}}}} \left( {\text{A}} \right)^{{2}} + \beta_{{{\text{BB}}}} \left( {\text{B}} \right)^{{2}} + \beta_{{{\text{CC}}}} \left( {\text{C}} \right)^{{2}} \\ \end{aligned}$$

A, B, and C are the independent factors; β_A_, β_B_, and β_C_ are linear coefficients; β_AB_, β_AC_ , and β_BC_ are cross product coefficients; and β_AA_, β_BB_, and β_CC_ are the quadratic coefficients. Y is the expected response, and the model intercept is denoted by β0.

To assess the equation model and make certain that the assumed values of every parameter were derived correctly, laboratory confirmation was carried out.

### Statistical methods for data analysis

Using Design-Expert 13 software, multiple linear regression analysis was performed on the acquired data .ANOVA was utilized to determine the statistically significant nature of the factors, and the F-value was computed at a probability *(p*-value) of below 0.05. To evaluate the model's validity, the multiple coefficients of correlation (*R*^*2*^) and the adjusted determination coefficient (*R*^*2*^*adj*) were computed. Also using Design-Expert 13 software, a three-dimensional graph was made to show the three most significant independent factors' contemporaneously effects on each response.

### Characterization of the agar prior and after biological treatment using FTIR analysis

The agar's functional groups were ascertained using the Fourier transform infrared spectroscopy (FTIR) method (IR, 8400s Shimadzu, Japan) with the IR fingerprints recorded between 4000 and 500 cm^−1^ using transmittance modes, both before and after biological treatment.

### Antioxidant activity (DPPH-free radical-scavenging assay)

After the optimization procedure, the fermentation hydrolysate's scavenging ability was evaluated using 1, 1-diphenyl-2-picrylhydrazyl (DPPH) via the process of free radical scavenging activity (RSA). A slightly altered technique that was previously reported was used to compare the RSA efficiency of the hydrolysate to ascorbic acid)^50^. In brief, 1 ml of 100 µM DPPH was combined with 400 µl of hydrolysate solutions. One milliliter of DPPH and 400 µl of 100% methanol were combined to create the negative control test. After different time (0, 15, 30 and 45 min) of dark storage in room temperature, and at a wavelength of 517 nm, the DPPH discolouration was observed.

The RSA scavenging efficiency was computed using:

$${\text{RSA}}\,\% = \left[ {\frac{{{\text{Control}}\,{\text{Absorbance}} - {\text{Sample}}\,{\text{Absorbance}}}}{{{\text{Control}}\,{\text{Absorbance}}}}} \right]*100$$

### Substrate substitution

#### Algae preparation

*Carolina officinalis* were collected from the eastern port of Alexandria, Egypt during September 2022. The collected algal biomass was washed with tap water to remove stones, sand and other undesirable impurities by distilled water, and dried at room temperature for 2 weeks^51,52^. The dried algal biomass was cut into small pieces, sieved to attain a particle size of 0.5–1.5 mm and stored in sealed plastic bags for further processing. For taxonomical identification according to, Mona et al.^53^ certain amounts of the collected seaweeds were stored in formalin (5%) in seawater, and this identification was then verified using the Algae Base website.

#### Algal biomass saccharification

After the optimization process of agarase production, agar in the final optimized medium was substituted with dried *Carolina officinalis* biomass at different concentration (w/v %) 2, 5. 10 & 15 for the agarase production using algae instead of agar as a substrate, where the fermentation optimization medium composed of: 1.645; peptone (g%), 24.494; yeast-extract (g%), Fe_2_SO_4_; 5 mg%, pH; 6, and a cultivation temperature of 30 °C to induce agarase production using *Bacillus subtilis* strain . The liberated reducing sugars through saccharification step was determined after 3 days incubation time by adding the cell free lysate to DNS reagent at equal ratio in a test tube and mixing vigorously. The developed red- brown color was measured at 540 nm. Afterwards, the sugar concentration was calculated as mg/ml according to the standard curve of glucose. Also, the agarase activity was recorded as described previously.

### Ethanol production

#### Preparation of algal biomass hydrolysate based medium for ethanol production

After saccharification step the cell free hydrolysate liquor was separated under aseptic condition by centrifugation (20 min, 5000 rpm), then sterilized, kept the pH at 5.0 (0.05 citrate buffer) and used for ethanol production. The freshly prepared overnight culture of yeast strain *Pichia pastoris* NRRL Y-11430 (Peoria IL, USA) in YPD broth was used for inoculation the ethanol production medium with 5% inoculum concentration. Flasks were incubated at 30 °C for 48 h, and then the ethanol concentrations were calculated according to Pourkarim et al.^54^. A control flask was prepared of YPD medium to compare the ethanol yields by using hydrolysate based medium against the synthetic YPD medium.

## Conclusion

The primary goal of this study was to statistically optimize agarase using non-marine isolate *Bacillus subtilis* PI utilizing agar as a carbon source, followed by determining the scavenging ability of agar-derived oligosaccharides. Finally, algal biomass was used instead of agar to produce bioethanol.

## Data Availability

All data produced during this study are included in this published article.
